# Overt GI bleeding from a Cameron lesion in an Ethiopian with NSAID use: Case report of an unusual condition

**DOI:** 10.1016/j.amsu.2022.103469

**Published:** 2022-03-04

**Authors:** Amir Sultan Seid, Eyouel Assefa Mamo

**Affiliations:** a Division of Gastroenterology, Department of Internal Medicine, Addis Ababa University, Addis Ababa, Ethiopia; b Senay Medical Center, Ethiopia

**Keywords:** Upper GI Bleeding, Cameron lesion, Endoscopy, Ethiopia, Africa

## Abstract

**Introduction:**

Cameron Lesions are linear breaks in the proximal gastric mucosa, mostly in association with hiatal hernia. The condition presents with chronic iron deficiency anemia and occasionally with obscure bleeding. Overt bleeding is very rare and has not been reported in sub- Saharan Africa context.

**Case:**

A 78 year old male patient, with an already diagnosed hiatal hernia and gouty arthritis, presented with massive upper GI bleeding requiring resuscitation and blood transfusion. The patient was taking indomethacin for a gout flare prior to the episode and clinical suspicion was a peptic ulcer disease as the culprit for the bleeding. Endoscopy was done and it showed two linear erosions with recent bleeding in the hernia sac. No other bleeding source was identified. The patient was treated with a Proton Pump Inhibitor (PPI).

**Discussion:**

Cameron lesions could present with massive bleeding and should be actively looked for in patients with hiatal hernia as they could be easily missed. Even with concurrent NSAID use, the condition could be a cause of major bleeding and careful evaluation is important. Management entails PPI therapy with occasional endoscopic intervention.

**Conclusion:**

In the setting of hiatal hernia, Cameron lesions should be actively looked for in patients presenting with overt GI bleeding.

## Highlights

• Cameron lesions are linear breaks in the proximal gastric mucosa in the setting of hiatal hernia.
• Usually these lesions present with chronic iron deficiency anemia.
• Overt bleeding from Cameron lesions is rare and has not been well described in Sub-Saharan Africa.
• Despite the use of NSAIDs, these lesions could be an alternative cause of major GI bleeding and should be actively sought.

## 1 Introduction

Cameron lesions are linear erosions in the proximal gastric mucosa, typically in the context of hiatal hernia. The condition was characterized in 1986 by Cameron & Higgins, and since then, there have been multiple reports of the condition [1]. Due to the difficulty in differentiating between erosions and ulcers, the term Cameron lesion is used as an umbrella term to encompass different patterns of presentations [2]. It is a relatively rare phenomenon, usually presenting as a cause of unexplained iron deficiency anemia. A recent systematic review identified 140 patients reported in the literature with females preferentially being affected [3]. Overt upper GI bleeding from the condition is unusual and is reported less frequently. In the Sub-Saharan Africa context, overt bleeding from Cameron lesions has not been reported well in the literature, and we report a case with such presentation. The case is reported in accordance with the 2020 SCARE guideline [4].

### 1.1 Case

A 78-year-old male presented to our emergency outpatient department with a one-day history of repeated vomiting of blood. Vomiting was described as initially being coffee ground matter and later mixed with clear blood. There was a passage of tarry stool of one episode prior to his presentation to the hospital.

The patient was diagnosed with gout previously and had been taking various Nonsteroidal Anti-inflammatory Drugs (NSAIDs) over the years. Prior to the week of his current presentation, the patient reported that he was taking Indomethacin for a “gout flare”.

He also has a previous diagnosis of hiatal hernia and a reflux disease made endoscopically three years ago. He was not taking intermittent Proton Pump Inhibitors (PPIs) during his current presentation.

Upon presentation to emergency, a voluminous vomiting of blood was witnessed by a healthcare professional. Initially, vital signs were remarkable for tachycardia (104 bpm), but Blood Pressure was normal (110/70 mmHg). Later, after the vomiting, BP dropped to 70/50 mmHg, and pulse rate rose to 118 bpm while temperature and oxygen saturation were within the normal limits.

Physical examination revealed pale conjunctivae and dark stool on Per rectal examination. No abdominal tenderness or mass was noted.

Investigations revealed that his hemoglobin was 8.9 gm/dl, and platelet count was 355,000/μl. Liver enzymes were normal, Creatinine was 1.1 mg/dl, and International Normalized Ratio was normal. The Stool Helicobacter Pylori Antigen test was negative, and an abdominal ultrasound was unremarkable. The patient was stabilized with a normal saline infusion, followed by two units of whole blood transfusion. He was also given an intravenous 40 mg esomeprazole.

**Fig. 1:**
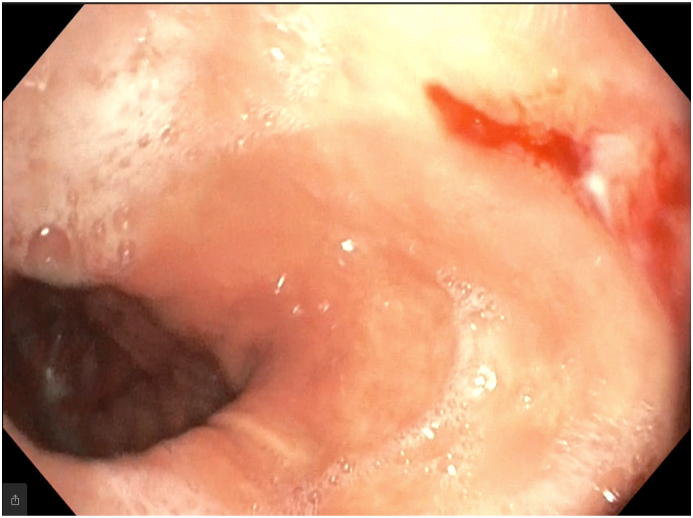
Forward view of a longitudinal erosion with recent bleeding in the hernia sac.

**Fig. 2:**
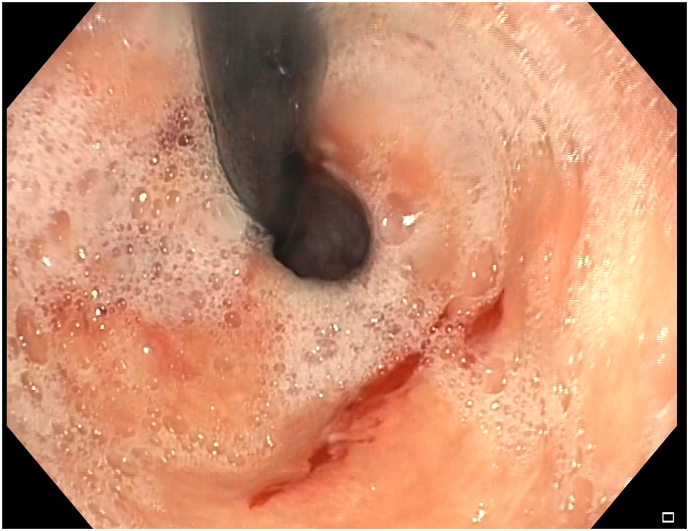
Retroflexed view of the erosion with signs of recent bleeding.

The next day, an endoscopic evaluation was done by a gastroenterologist at a large endoscopy center, with a presumptive assessment of peptic ulcer bleeding. It showed a large hiatal hernia with two erosions located inside the hernia sac with features of recent bleeding. Lesions were linear, oriented in the longitudinal axis of the gastric folds[ Fig. 1, Fig. 2]. There was no active bleeding noted, and no retained blood was present in the stomach. No ulcer was seen in the duodenum. The patient was treated with PPI therapy, and after transfusion, his hemoglobin rose to 11.5 mg/dl. No bleeding recurred, and the patient's clinical condition significantly improved. He was put on oral Omeprazole 20 mg orally twice per day and referred to a surgeon for the management of the large hiatal hernia.

## 2 Discussion

Cameron lesions are caused by linear breaks in the stomach's mucosa, usually in a hiatal hernia sac. The underlying reason for the development of the lesions is mechanical trauma by the diaphragm edge to the hernia sac, causing local ischemia on the gastric folds. Histologic evaluation of the findings has shown infiltration by inflammatory cells and superficial alteration [5].

Typically patients present with an unexplained iron deficiency anemia, requiring iron therapy. Larger Hiatal hernias are associated with more prevalence of anemia. In addition, a direct relationship has been demonstrated with the size of hiatal hernia risk of Cameron lesions [6]. Overt massive upper GI bleeding from Cameron lesions has been reported, albeit less frequently than occult & obscure bleeding [7]. In a large review of 3960 patients with major upper GI bleeding at the UCLA over 17 years, Cameron lesions were a culprit in only 0.6% of the cases [8]. The review was done in a well-equipped facility with more experienced endoscopists, and it points to the possibility of the lesions being missed in less developed setups.

Diagnosis of Cameron lesions is made usually by an endoscopy. In the original paper, Cameron described the lesions as “typically white, narrow and elongated with the longitudinal axis corresponding to the longitudinal direction of the gastric mucosal folds”, which is consistent to the description in our index case [1]. Endoscopists could miss these lesions as they are located in difficult-to-spot areas. According to a recent systematic review of the condition, almost two third of the patients with the lesions had a prior endoscopic evaluation where diagnosis had not been made [3].

Most patients with Cameron lesions are treated with medical therapy, which entails oral acid suppression and iron supplementation. Some patients are treated with a surgical approach, usually using a fundoplication procedure to address the underlying hiatal hernia. Endoscopic intervention is sometimes employed in the management of the lesions, with marginal benefit. In our case, the patient was clinically stable by the time the endoscopic procedure was done, and there was no active bleeding or stigmata of high risk rebleeding. A judgment to utilize endoscopic intervention is made based on the overall patient situation and endoscopist's experience, as the location of such lesions might make interventions technically difficult. In case intervention is required, an injection with epinephrine can be used to arrest active bleeding. Alternatively, endoclips can be deployed in lesions with active bleeding or high risk stigmata of rebleeding [8]. A previous case report has also described successful usage of band ligation in a Cameron lesion with a visible vessel [9].

In Sub-Saharan Africa, massive bleeding from Cameron lesions has not been well characterized previously. There are reports of the condition associated with anemia, but such overt presentation has not been described [10]. As this condition tends to be overlooked or misrepresented usually, a careful evaluation is important to make a proper diagnosis. Emphasis is on portal hypertension and ulcer-related bleeding in the evaluation of upper GI bleeding in the continent, but keeping an open eye for such lesions is important, mainly in the context of hiatal hernia [11]. The index case shows that Cameron lesions could be a culprit of significant overt upper GI bleeding even in the context of NSAIDs intake and should be looked for actively in patients with hiatal hernia.

## Provenance and peer review

Not commissioned, externally peer-reviewed.

## Ethical approval

As this is a case report, ethical approval is not required in our institutions.

## Sources of funding

No funding to declare.

## Author contribution

AS did the endoscopic evaluation of the patient, did the literature review and manuscript development. EM did the clinical evaluation and management of the patient, did part of the manuscript development.

## Consent

Written informed consent was obtained from the patient for publication of this case report and accompanying images. A copy of the written consent is available for review by the Editor-in-Chief of this journal on request.

## Registration of research studies

1. Name of the registry:

2. Unique Identifying number or registration ID:

3. Hyperlink to your specific registration (must be publicly accessible and will be checked):

## Guarantor

Amir Sultan Seid (MD).

## Declaration of competing interest

No Conflict of Interest to declare.
